# Oral pathogens exacerbate Parkinson’s disease by promoting Th1 cell infiltration in mice

**DOI:** 10.1186/s40168-023-01685-w

**Published:** 2023-11-17

**Authors:** Xue-Bing Bai, Shuo Xu, Lu-Jun Zhou, Xiao-Qian Meng, Yu-Lin Li, Yan-Lin Chen, Yi-Han Jiang, Wen-Zhen Lin, Bo-Yan Chen, Lin-Juan Du, Guo-Cai Tian, Yan Liu, Sheng-Zhong Duan, Ya-Qin Zhu

**Affiliations:** 1 Department of General Dentistry, Shanghai Ninth People’s Hospital, Shanghai Jiao Tong University School of Medicine, 639 Zhizaoju Road, Shanghai, 200011 China; 2 College of Stomatology, Shanghai Jiao Tong University, 639 Zhizaoju Road, Shanghai, 200011 China; 3 National Center for Stomatology, 639 Zhizaoju Road, Shanghai, 200011 China; 4 National Clinical Research Center for Oral Diseases, 639 Zhizaoju Road, Shanghai, 200011 China; 5 Shanghai Key Laboratory of Stomatology, 639 Zhizaoju Road, Shanghai, 200011 China; 6 Shanghai Research Institute of Stomatology, 639 Zhizaoju Road, Shanghai, 200011 China; 7 Laboratory of Oral Microbiota and Systemic Diseases, Shanghai Ninth People’s Hospital, Shanghai Jiao Tong University School of Medicine, 639 Zhizaoju Road, Shanghai, 200011 China; 8 Department of Periodontology, Shanghai Ninth People’s Hospital, Shanghai Jiao Tong University School of Medicine, 639 Zhizaoju Road, Shanghai, 200011 China

**Keywords:** Parkinson’s disease, Periodontitis, Oral pathogens, T helper 1 cells

## Abstract

**Background:**

Parkinson’s disease (PD) is a common chronic neurological disorder with a high risk of disability and no cure. Periodontitis is an infectious bacterial disease occurring in periodontal supporting tissues. Studies have shown that periodontitis is closely related to PD. However, direct evidence of the effect of periodontitis on PD is lacking. Here, we demonstrated that ligature-induced periodontitis with application of subgingival plaque (LIP-SP) exacerbated motor dysfunction, microglial activation, and dopaminergic neuron loss in 1-Methyl-4-phenyl-1,2,3,6-tetrahydropyridine (MPTP)-induced PD mice.

**Results:**

The 16S rRNA gene sequencing revealed that LIP-SP induced oral and gut dysbiosis. Particularly, *Veillonella parvula* (*V. parvula*) and *Streptococcus mutans* (*S. mutans*) from oral ligatures were increased in the fecal samples of MPTP + LIP-SP treated mice. We further demonstrated that *V. parvula* and *S. mutans* played crucial roles in LIP-SP mediated exacerbation of motor dysfunction and neurodegeneration in PD mice. *V. parvula* and *S. mutans* caused microglial activation in the brain, as well as T helper 1 (Th1) cells infiltration in the brain, cervical lymph nodes, ileum and colon in PD mice. Moreover, we observed a protective effect of IFNγ neutralization on dopaminergic neurons in *V. parvula-* and *S. mutans-*treated PD mice.

**Conclusions:**

Our study demonstrates that oral pathogens *V. parvula* and *S. mutans* necessitate the existence of periodontitis to exacerbate motor dysfunction and neurodegeneration in MPTP-induced PD mice. The underlying mechanisms include alterations of oral and gut microbiota, along with immune activation in both brain and peripheral regions.

Video Abstract

**Supplementary Information:**

The online version contains supplementary material available at 10.1186/s40168-023-01685-w.

## Introduction

Parkinson’s disease (PD) is the second most prevalent form of neurodegenerative disorders, affecting 2% of the global population over the age of 60 [1]. The main pathological feature of PD is the loss of dopaminergic neurons in the substantia nigra, which can lead to motor symptoms [2]. Inflammation plays an important role in PD, and microglia are activated and secrete inflammatory mediators that impair the central nervous system (CNS), leading to neurodegeneration [3, 4].

Periodontitis is the most common chronic inflammatory disease in adults [5] and mainly involves complex dynamic interactions between periodontal pathogens and host immune responses [6]. Periodontitis can cause low-grade systemic inflammation and has systemic effects [7]. Furthermore, periodontal pathogens can invade other organs and induce an immune response. Therefore, periodontitis has negative effects on some chronic systemic diseases such as cardiovascular disease, diabetes, and rheumatoid arthritis [8–10]. Recent studies have shown that systemic inflammation caused by periodontitis can induce neuroinflammation by damaging blood‒brain barrier permeability, activating microglia, and facilitating immune cell invasion into the brain [11–13]. These results suggest that periodontitis may have an effect on PD.

Several clinical studies have shown a correlation between periodontitis and PD. A retrospective cohort study showed that individuals who were newly diagnosed with periodontitis had a higher risk of developing PD than those without periodontitis [14]. Another study has revealed a positive correlation between increased tooth loss and new cases of PD [15]. Moreover, dental plaque microbiota is related to PD motor symptoms severity [16]. And dental scaling has been found to reduce the risk of PD [17]. Specifically, individuals aged 40–69 years without periodontitis show a protective effect of dental scaling against PD development, particularly when performed for over five consecutive years. In contrast, patients aged ≥ 70 years with discontinued scaling (not maintained for five consecutive years) demonstrate an increased risk of PD [17]. Furthermore, local periodontal inflammation is associated with neuroinflammation of PD [18]. Oral pathogens may play critical roles in the aggravation of PD by periodontitis. A key pathogen in periodontitis is *Porphyromonas gingivalis* (*P. gingivalis*), which plays an important role in exacerbating dopaminergic neuron loss in PD [19]. Studies have shown that oral pathogens can cause neurodegeneration by directly invading the brain [20] or by ectopically colonizing in other organs and causing systemic inflammation [19]. However, there is no direct evidence that periodontitis exacerbates PD.

In this study, we hypothesized that in the presence of periodontitis, oral pathogens could exacerbate PD symptoms by triggering peripheral and brain inflammatory responses. We used a periodontitis mouse model combined with an MPTP-induced PD mouse model to investigate how oral pathogens affected PD. Furthermore, we investigated the mechanisms by assessing the ectopic colonization of oral pathogens and their influence on systemic immunity.

## Materials and methods

### Animals and treatments

Eight-week-old male C57BL/6 mice (weight, 22–25 g) from Beijing Vital River Laboratories (Beijing, China) were maintained (5 mice/cage, 12-h light/dark cycle) in a specific-pathogen-free (SPF) environment at 22 ± 2.0℃ with free access to food and water. One week of acclimatization was required before the experiment. All research performed on mice was approved by the Institutional Animal Care and Use Committee (IACUC) of Cyagen Biosciences, China (Document No. ACU21-A039). Ethical approval for collection of subgingival plaques from periodontitis patients was obtained from the Institutional Review and Ethics Board of Shanghai Ninth People’s Hospital, Shanghai Jiao Tong University School of Medicine (No. SH9H-2018-T66–3).

1-Methyl-4-phenyl-1,2,3,6-tetrahydropyridine (MPTP) (Sigma‒Aldrich) was intraperitoneally (i.p.) injected into the mice (15 mg/kg, dissolved in saline) for 5 consecutive days to induce PD.

In the ligature-induced periodontitis with application of subgingival plaque (LIP-SP) treatment experiment, the LIP-SP model was established to induce periodontitis as described in previous studies [21, 22]. Mouse maxillary second molars were ligated with 5–0 silk to establish ligature-induced periodontitis (LIP). Subgingival plaque (SP) samples from 50 patients with periodontitis were mixed and divided into 50 aliquots. The SP samples were centrifuged and resuspended in 500 µL of 2% carboxymethylcellulose (CMC) and then applied to the entire gingival tissue of maxillary molar teeth (50 μL per mouse) on the day after LIP, 3 times per week for 4 weeks. The mice were randomly divided into 4 groups: the control group (2% CMC and normal saline were used as controls for SP and MPTP, respectively), LIP-SP group (normal saline was used as a control for MPTP), MPTP group (2% CMC was used as a control for SP), and MPTP + LIP-SP group.

In the ligature-induced periodontitis and oral infection of *Veillonella parvula* and *Streptococcus mutans* mixture (LIP-mix) treatment experiment, we established a LIP-mix model to investigate the effects of *Veillonella parvula* (*V. parvula*) and *Streptococcus mutans* (*S. mutans*) on PD. In brief, the mice were first subjected to LIP and then orally inoculated *V. parvula* and *S. mutans* mixture (mix). A total of 1*10^9^ colony-forming units of live *V. parvula* and *S. mutans* were mixed and centrifuged at 5000 r for 5 min. Each pellet was resuspended in 50 μL of 2% CMC and then applied to the entire gingival tissue of maxillary molar teeth (50 μL/mouse) on the day after LIP [23], 3 times per week for 4 weeks. In this section, the mice were randomly divided into 4 groups: the control group (2% CMC and normal saline were used as controls for *V. parvula* and *S. mutans* and MPTP, respectively), LIP-mix group (normal saline was used as a control for MPTP), MPTP group (2% CMC was used as a control for *V. parvula* and *S. mutans*), and MPTP + LIP-mix group.

In the IFNγ neutralization experiment, the mice were intraperitoneally injected with 250 μg of anti-IFNγ (BioXcell, BE0054) or IgG (BioXcell, BE0094) in 200 μL of PBS on Day -1 and Day 3 after MPTP injection. The mice were randomly divided into 4 groups: the MPTP group (2% CMC was used as the control for *V. parvula* and *S. mutans*, and IgG was used as the control for anti-IFNγ), MPTP + anti-IFNγ group (2% CMC was used as the control for *V. parvula* and *S. mutans*), MPTP + LIP-mix group (IgG was used as the control for anti-IFNγ), and MPTP + LIP-mix + anti-IFNγ group.

### Behavioral tests

All behavioral tests were performed on the 1st day after the last MPTP or saline exposure. There was at least a 30-min interval between trials.

To test motor mediation and balance impairment in mice, a rotarod test was conducted [24, 25]. The mice were placed on an accelerating rotarod. During behavioral training, the rotation speed of the rotarod remained at 10 rpm for 5 min. During the formal test, the rotarod speed was initially set at 4 rpm and then gradually increased to 40 rpm over 2 min, and the time for the animal to drop was recorded.

The traction test is mainly used to measure muscle strength and balance in PD mice [26]. The mice were placed on a metal wire (1 mm in diameter) such that both forelimbs grasped the wire, and the hind limbs were suspended. The score was 4 points if the mice grasped the wire with four limbs, 3 points if three limbs grasped the wire, 2 points if only both forelimbs grasped the wire, 1 point if one forelimb grasped the wire, and 0 points if no limb grasped the wire.

### Strains and culture media

*V. parvula* (ATCC 10790) was cultured in reinforced clostridial broth (HLD137, Haling Biological Technology Co., Ltd), and 1.5 mL of 60% DL-lactate (Sigma‒Aldrich) was added per 100 mL of broth. *S. mutans* UA159 was cultured in brain heart infusion (BHI, Bacto™ brain heart infusion) broth. All strains were grown under anaerobic conditions at 37°C for 24 h.

### Western blot analysis

Proteins were extracted from the mouse striatum with RIPA buffer and a protease inhibitor cocktail (Biotool, B14001). Equal amounts of protein were subsequently denatured in 4 × loading buffer and separated with a 10% SDS‒PAGE gel. The antibodies used were anti-tyrosine hydroxylase (1:1000, #58844S, Cell Signaling Technology) and anti-β-actin (1:5000, 66,009–1-Ig, Proteintech). The relative density of each protein was quantified by ImageJ v1.51 software.

### Immunohistochemistry (IHC)

The mice were anesthetized with isoflurane and perfused with saline through the heart. The brains were removed, fixed, dehydrated, embedded in OCT (Optimal cutting temperature compound) and cut into 40 μm coronal sections. The sections were stained with an immunohistochemical detection kit (PK10006, Proteintech). The antibody used was anti-tyrosine hydroxylase (1:100, #58844S, Cell Signaling Technology). The number of TH-positive neurons in the substantia nigra (SN) and the optical density of the TH-positive area in the striatum (ST) were measured using ImageJ v1.51 software.

### Immunofluorescence analysis

Brain sections containing the ST and SN were blocked in PBS with 0.1% Triton X-100 and 5% goat serum at 25°C for 1 h. Then, the sections were incubated with primary antibodies at 4°C overnight. Subsequently, the sections were washed with PBS and stained with Alexa 488- or 594-conjugated secondary antibodies for 2 h at room temperature. The antibodies used were mouse anti-tyrosine hydroxylase (1:100, #45648S, Cell Signaling Technology) and rabbit anti-Iba1 (1:1000, Wako). The number of cells in the SN and ST were measured using ImageJ v1.51 software.

### Oral and gut genomic DNA extraction and 16S-rRNA sequencing

Oral microbiome was evaluated in oral ligatures. In the MPTP group, oral ligatures were collected from mice 3 h post ligature placement [27]. Gut microbiome was evaluated in feces. Total genomic DNA was extracted from oral ligatures, brain tissue and feces using the OMEGA Soil DNA Kit (Omega Bio-Tek). Then, single-molecule real-time sequencing was performed with the PacBio Sequel platform at Shanghai Personal Biotechnology Co., Ltd.

16S rRNA analysis was performed using QIIME2 2019.4 (PeerJ Preprints, 6, e27295v2.). Sequence data analyses were mainly performed with R packages (v3.2.0) and QIIME2. ASV-level alpha diversity indices were calculated using the ASV table in QIIME2 and visualized as box plots. Jaccard metrics were used to conduct beta diversity analysis and research the structural variations in microbial communities among samples. The beta diversity is shown as a principal coordinate analysis (PCoA). Random forest analysis was performed using QIIME2 with default settings to distinguish the samples from different groups.

### Quantitative real-time PCR

Quantitative real-time PCR (qRT‒PCR) was performed with TB Green PCR Master Mix (Thermo Fisher Scientific) on a LightCycler 480 II system (Roche Diagnostics). The reference gene was 16S. The primers are as follows: 16S primer, forward 5’- ACTCCTACGGGAGGCAGCAGT-3’, reverse 5’- GTATTACCGCGGCTGCTGGCAC-3’; *V. parvula* primer, forward 5’-TGAAAGGTGGCCTCTATTTAT-3’, reverse 5’- CAATCCTTCTAACTGTTCGCAAG-3’; and *S. mutans* primer, forward 5’- ACTACACTTTCGGGTGGCTTG-3’, reverse 5’-CAGTATAAGCGCCAGTTTCATC-3’.

### Flow cytometry

Single-cell suspensions of brain, cervical lymph node, ileum and colon tissue were centrifuged and stained with Live/Dead Fixable Viability Stain 510, incubated with anti-CD16/32 antibodies and then stained with surface antibodies to detect cell surface antigens. To detect intracellular cytokines, the cells were incubated with a cell activation cocktail (BioLegend) for 4–6 h and then stained with surface antibodies. After that, the cells were fixed and permeabilized by using an intracellular cytokine staining kit (eBioscience) to detect IFNγ and IL-17A expression. The antibodies used for staining were anti-CD45 (557,659, BD Pharmingen), anti-B220 (553,090, BD Pharmingen), anti-CD3e (553,061, BD Pharmingen), anti-CD4 (550,954, BD Pharmingen), anti-CD8 (553,035, BD Pharmingen), anti-CD11b (550,993, BD Pharmingen), anti-IL-17A (506,903, Biolegend), and anti-IFNγ (505,830, Biolegend).

### Statistical analysis

The data were analyzed with Prism (GraphPad Software, CA) by two-way ANOVA with Tukey’s multiple comparison test or unpaired Student’s t test. The data are presented as the mean ± SEM, and values of *P* < 0.05 were considered statistically significant.

## Results

### Ligature-induced periodontitis with application of subgingival plaque (LIP-SP) exacerbates motor dysfunction and dopaminergic neuronal loss in MPTP-induced PD mice

**Fig. 1:**
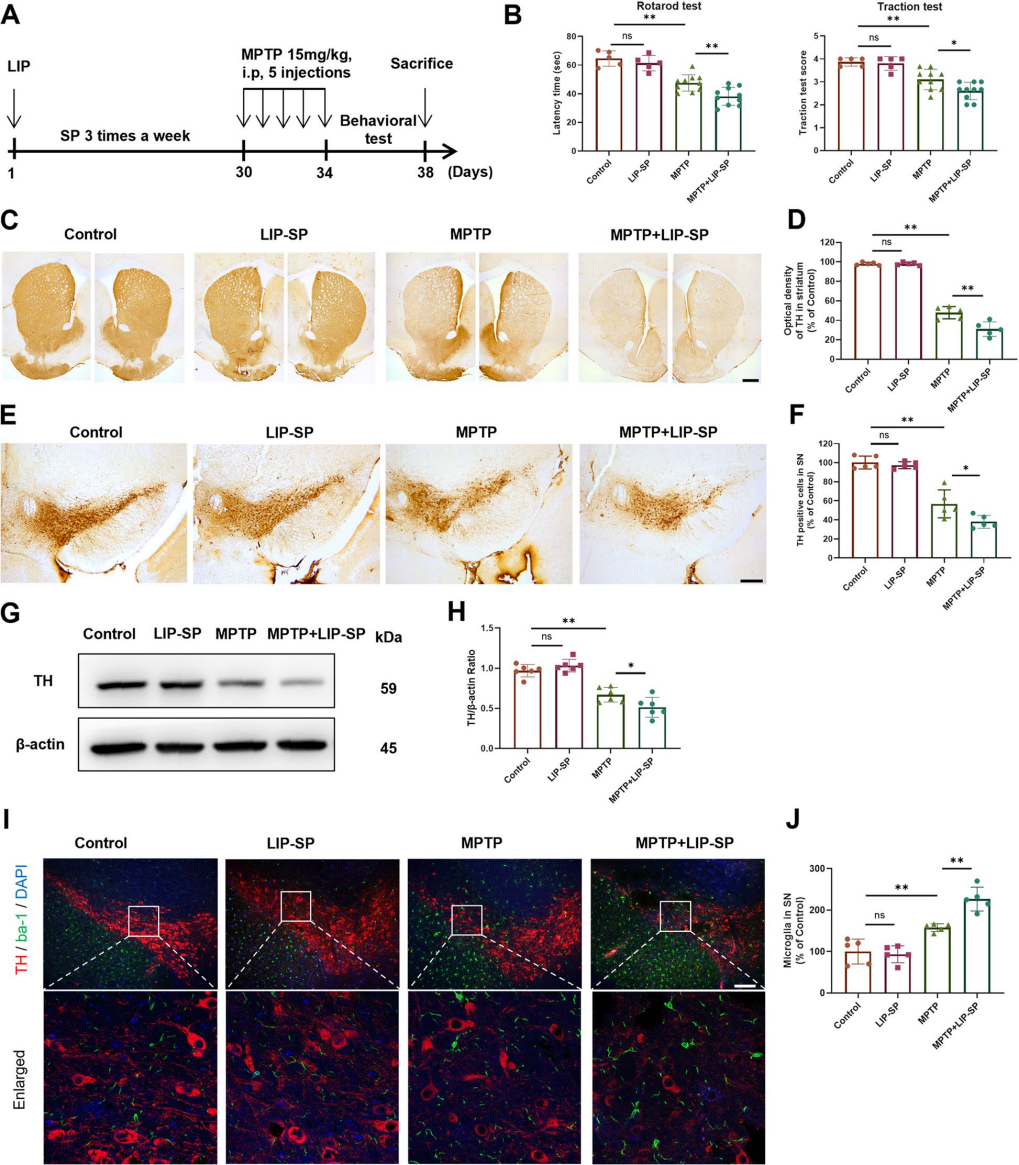
LIP-SP exacerbates motor dysfunction and dopaminergic neuronal loss in MPTP-induced PD mice. **A** Schematic illustration of the experimental procedure. MPTP, 1-methyl-4-phenyl-1,2,3,6-tetrahydropyridine. LIP, ligature-induced periodontitis. SP, subgingival plaque. LIP-SP, ligature-induced periodontitis with application of subgingival plaque. **B** Bar plots of performance in behavioral tests, including rotarod test and traction test. *n* = 5:5:10:10. **C**-**D** Representative immunohistochemical staining (**C**) and quantification (**D**) of tyrosine hydroxylase (TH)-positive fibers in striatum (ST). *n* = 5:5:5:5. **E**–**F** Representative immunohistochemical staining (**E**) and quantification (**F**) of TH-positive neurons in substantia nigra (SN). *n* = 5:5:5:5. Scale bar: 200 μm. **G** Western blotting analysis and quantification (**H**) of TH in ST. *n* = 5:5:5:5. **I** Representative immunofluorescence staining of dopaminergic neuron marker TH (red) and microglia marker Iba-1 (green) in SN. DAPI: 4’,6-diamidino-2-phenylindole dihydrochloride. **J** Quantitative analysis of the number of microglia in SN. *n* = 5:5:5:5. Scale bar: 200 μm.Values are expressed as mean ± SEM (standard error of the mean). Two-way ANOVA followed by Tukey’s multiple comparison test was used for statistical analysis. ns, not significant. *P < 0.05, **P < 0.01

To assess the effects of periodontitis on motor function and dopaminergic neuron loss in MPTP-induced PD mice, we first performed LIP and applied SP three times per week to induce periodontitis (Fig. S1). After one month, we induced PD in the mice by an intraperitoneal injection of MPTP (Fig. 1A). Behavioral tests showed that MPTP significantly decreased the latency time in the rotarod test and the traction test score compared with those in the control group (Fig. 1B). LIP-SP impaired motor function in the rotarod test and traction test in MPTP-induced PD mice but had no effect on control mice (Fig. 1B).

Loss of dopaminergic neurons in the striatum (ST) and substantia nigra (SN) is the main pathological characteristic of PD, and tyrosine hydroxylase (TH) is a rate-limiting enzyme that synthesizes dopamine and is used as a marker of dopaminergic neurons [28]. We examined the effect of LIP-SP on the impairment of dopaminergic neurons by characterizing TH expression in the ST and SN. As expected, IHC showed that MPTP administration caused a nearly 50% reduction in TH-positive fibers in the ST (Fig. 1C-D) and TH-positive dopaminergic neurons in the SN (Fig. 1E-F). Importantly, MPTP-LIP-SP resulted in a robust reduction in dopaminergic neurons in the ST (Fig. 1C-D) and the SN (Fig. 1E-F) compared with those in the MPTP group. Consistent with this observation, western blot analysis showed a significant decrease in the TH protein levels in the ST of MPTP-induced PD mice compared with control mice. LIP-SP exacerbated TH protein loss after MPTP treatment (Fig. 1G-H). LIP-SP did not significantly affect the levels of dopaminergic neurons in control mice without MPTP-induced PD (Fig. 1C-H). In addition, immunofluorescence analysis showed that LIP-SP accelerated microglial activation in the SN of MPTP-induced PD mice (Fig. 1I-J). Neither LIP nor SP alone aggravated dopaminergic neuronal loss in MPTP-induced PD mice (Fig. S2), suggesting that the exacerbation of PD requires the conjunction of oral pathogens with periodontitis.

### LIP-SP alters the oral and gut microbiota of MPTP-induced PD mice

**Fig. 2:**
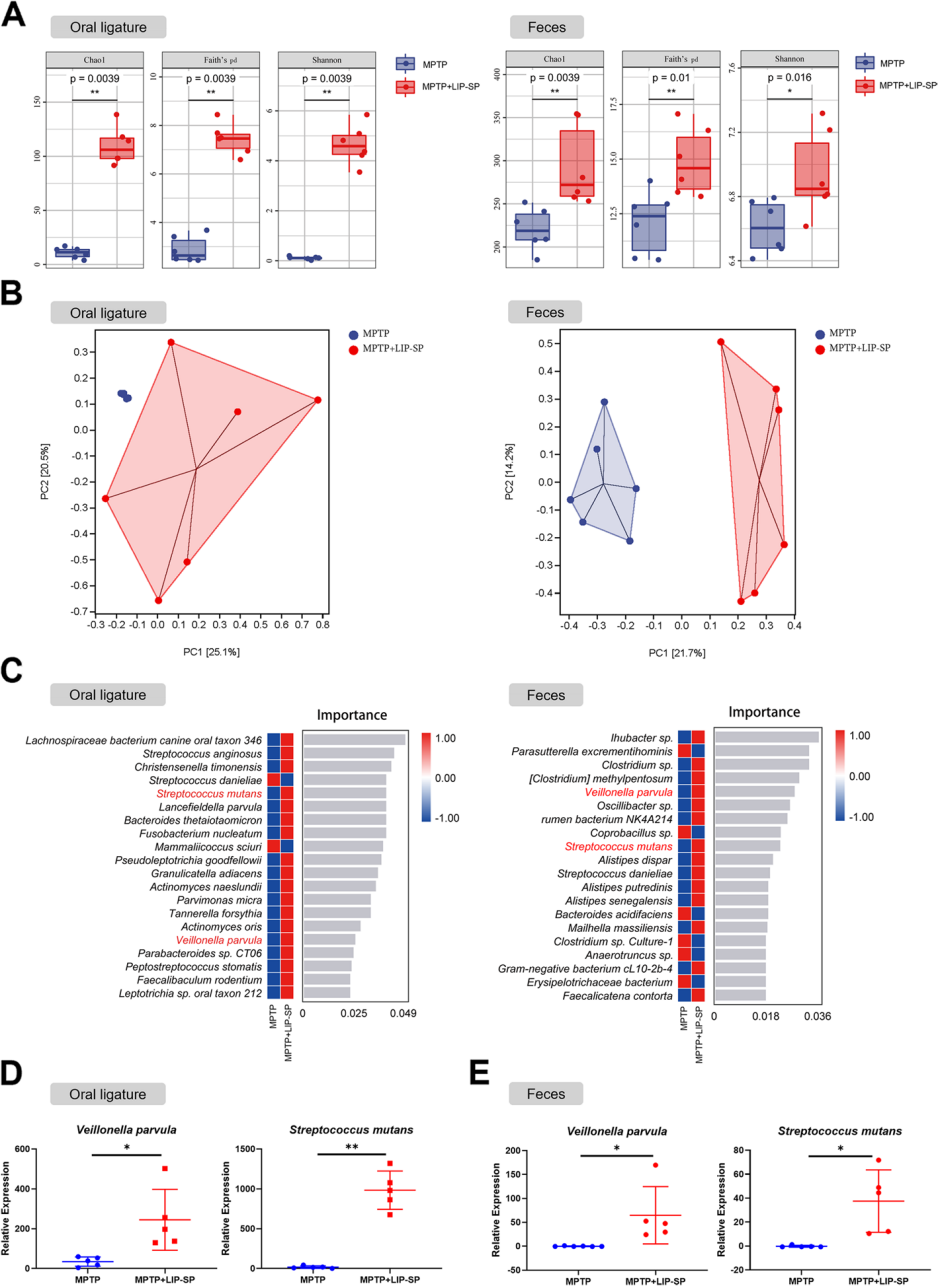
LIP-SP alters both the oral and gut microbiota of MPTP-induced PD mice. **A** Alpha diversity of oral and gut microbiota assessed by Chao 1, Faith’s phylogenetic diversity (Faith’s pd), and Shannon index. 16S rRNA gene sequencing was used to analyze the microbiota in oral ligatures and feces of mice. **B** Principal coordinate analysis (PCoA) of oral and gut microbiota. **C** Random forest analysis of oral and gut microbiota at the species level. **D** Quantitative real-time polymerase chain reaction (qRT-PCR) analysis of *Veillonella parvula* and *Streptococcus mutans* in oral ligatures.* n* = 5:5. **E** qRT-PCR analysis of *Veillonella parvula* and *Streptococcus mutans* in feces.* n* = 5:5. Data are presented as mean ± SEM. Student’s t-test was used for statistical analysis. *P < 0.05, **P < 0.01

Previous studies have demonstrated the role of gut dysbiosis in PD, and we hypothesized that LIP-SP could affect the pathogenesis of PD through the oral-gut axis. We analyzed the oral ligatures and feces of mice by 16S rRNA gene sequencing. Alpha diversity analysis showed a significant difference in Faith’s phylogenetic diversity (Faith’s pd) in oral and gut samples from the MPTP group and the MPTP + LIP-SP group. The diversity of the oral and gut microbiomes in MPTP + LIP-SP treated mice were obviously higher than those in MPTP-treated mice (Fig. 2A). Beta diversity analysis showed significant differences in the oral and gut microbial composition between the MPTP group and the MPTP + LIP-SP group (Fig. 2B). However, in the brain, neither alpha diversity nor beta diversity was significantly different between the two group (Fig. S3A-B).

Next, we used random forest analysis to identify potential pathogenic bacteria. The oral pathogens *Veillonella parvula* (*V. parvula*) and *Streptococcus mutans* (*S. mutans*) were enriched in the oral ligatures and feces of MPTP + LIP-SP treated mice compared with MPTP treated mice (Fig. 2C). However, we did not detect any oral bacteria invading the brain in the MPTP + LIP-SP group (Fig. S3C). Next, we used qRT-PCR to evaluate the DNA levels of *V. parvula* and *S. mutans* in the oral ligatures and feces. Compared with those in the MPTP group, the DNA levels of *V. parvula* and *S. mutans* were notably increased in the oral ligatures and feces of the MPTP + LIP-SP group (Fig. 2D-E).

### Ligature-induced periodontitis and oral infection of *V. parvula *and *S. mutans* mixture (LIP-mix) exacerbates MPTP-mediated motor dysfunction and dopaminergic neuronal loss

**Fig. 3:**
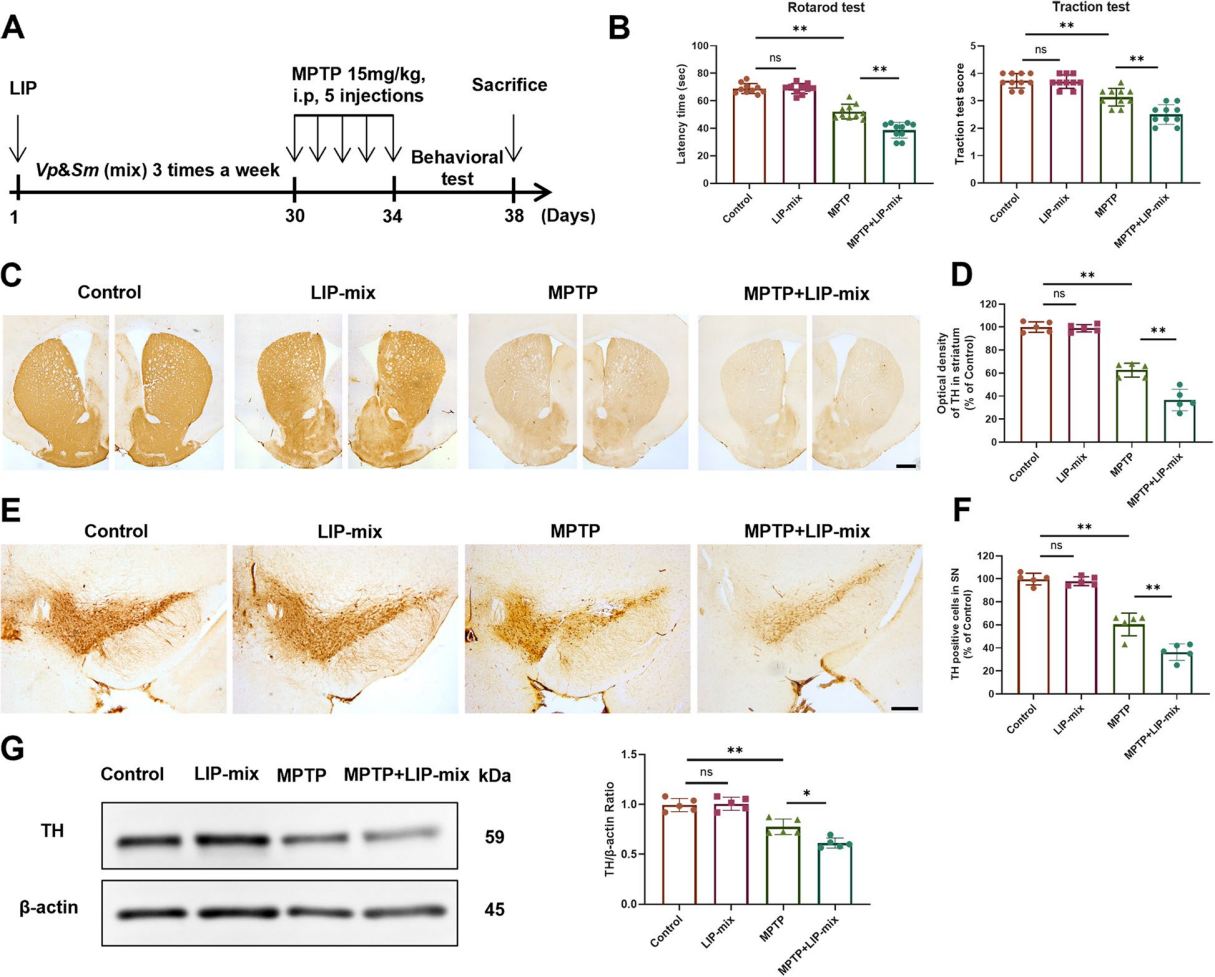
LIP-mix aggravates MPTP-induced motor dysfunction and dopaminergic neuronal loss. **A** Schematic illustration of the experimental procedure. **B** Bar plots of performance in behavioral tests, including rotarod test and traction test. Mix: *V. parvula* and *S. mutans* mixture. LIP-mix: ligature-induced periodontitis and oral infection of *V. parvula* and *S. mutans* mixture.* n* = 10:10:10:10. **C-D** Representative immunohistochemical staining (**C**) and quantification (**D**) of TH-positive fibers in ST. *n* = 5:5:5:5. **E–F** Representative immunohistochemical staining (**E**) and quantification (**F**) of TH-positive neurons in SN. *n* = 5:5:5:5. Scale bar: 200 μm. **G** Western blotting analysis of TH in ST. *n* = 5:5:5:5. Values are expressed as mean ± SEM. Two-way ANOVA followed by Tukey’s multiple comparisons test was used for statistical analysis. ns, not significant. *P < 0.05, **P < 0.01

We then investigated the effect of *V. parvula* and *S. mutans* on MPTP-induced PD. LIP-mix mouse model was established by conducting LIP and followed by application of *V. parvula* and *S. mutans* mixture (mix) three times per week for one month (Fig. S1), and LIP-mix induced more severe alveolar bone resorption than LIP (Fig. S1); MPTP was then injected to induce PD (Fig. 3A). As expected, intraperitoneal injection of MPTP impaired motor function, as determined by behavioral tests. In the MPTP + LIP-mix group, the latency time in the rotarod test was shortened, and the score of the traction test was significantly decreased compared with those in the MPTP group (Fig. 3B). LIP-mix did not affect motor function in the absence of intraperitoneal injection of MPTP (Fig. 3B).

Furthermore, LIP-mix reinforced MPTP-induced dopaminergic neuronal damage in the ST and SN. The numbers of dopaminergic fibers and neurons in the ST and SN were significantly decreased after MPTP injection, and LIP-mix treatment caused a further loss of dopaminergic neurons in the ST and SN of MPTP-induced PD mice (Fig. 3C-F). The level of TH positive protein in the ST also showed a similar trend (Fig. 3G). However, there was no significant difference in the levels of dopaminergic fibers and neurons between the control group and LIP-mix group (Fig. 3C-G). Then we conducted additional experiments in which mice were orally administered *V. parvula* and *S. mutans* without LIP induction, and then induced PD using MPTP (Fig. S4A). We found that without LIP these two bacteria scarcely colonized in the gut (Fig. S4B) and did not exacerbate PD (Fig. S4C). These data suggest that *V. parvula* and *S. mutans* require the presence of periodontitis to colonize the mouse gut and exert an exacerbating effect on PD.

### LIP-mix stimulates MPTP-induced microglial activation and CD4^+^ T cell infiltration in the mouse brain

**Fig. 4:**
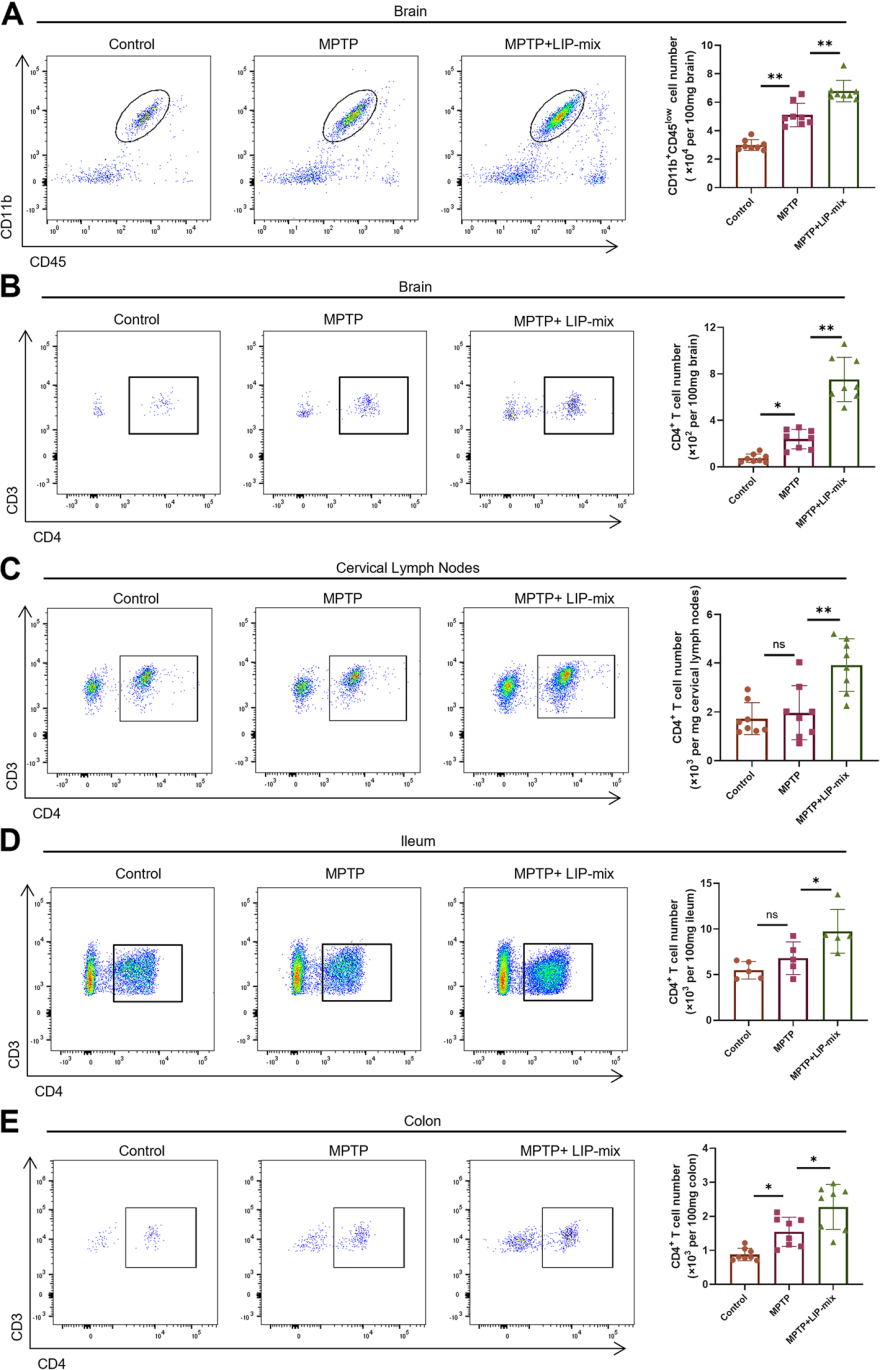
LIP-mix stimulates MPTP-induced microglial activation and CD4^+^ T cell infiltration in mouse brain. **A** Representative flow cytometry analysis and quantification of CD45^low^ CD11b^+^ microglia in mouse brain. **B-E** Representative flow cytometry analysis and quantifications of CD3^+^ CD4.^+^ T cells in mouse brain (**B**), cervical lymph nodes (**C**), ileum (**D**), and colon (**E**). *n* = 5–8 per group. Values are expressed as mean ± SEM. Two-way ANOVA followed by Tukey’s multiple comparisons test. ns, not significant. *P < 0.05, **P < 0.01

To investigate neuroinflammation in the brain, we analyzed brain tissue samples by flow cytometry. The number of microglia (CD45^low^ CD11b^+^) and CD4^+^ T cells (CD3^+^ CD4^+^) was prominently increased in the MPTP group compared with the control group (Fig. 4A, B). Notably, LIP-mix treatment further increased the number of microglia and CD4^+^ T cells in the brains of MPTP-induced PD mice (Fig. 4A, B).

Next, we examined immune cells in the cervical lymph nodes, ileum and colon by flow cytometry. CD4^+^ T cells were dramatically increased in the cervical lymph nodes of the MPTP + LIP-mix group compared with the MPTP group, although there was no difference between the control group and the MPTP group (Fig. 4C). Similarly, LIP-mix increased the number of CD4^+^ T cells in the MPTP + LIP-mix group compared with the MPTP group (Fig. 4D). Notably, the number of CD4^+^ T cells in the colon was elevated in the MPTP group compared with the control group, and this was further increased by LIP-mix in MPTP + LIP-mix group (Fig. 4E).

### LIP-mix promotes the accumulation of Th1 cells in MPTP-induced PD mice

**Fig. 5:**
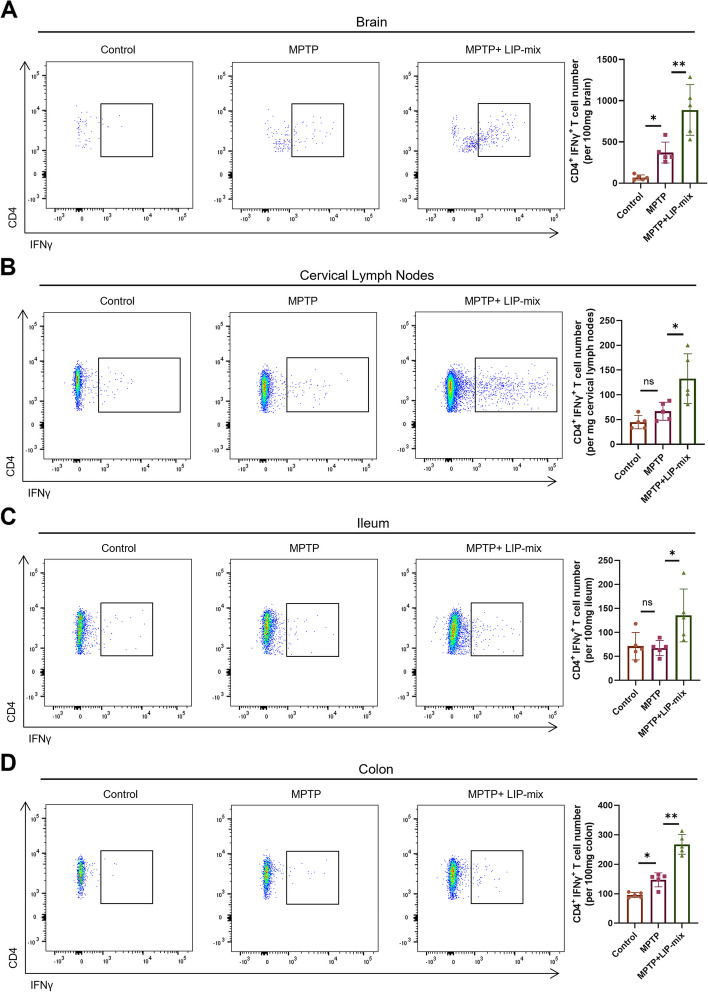
LIP-mix promotes the accumulation of Th1 cells in MPTP-induced PD mice. Representative flow cytometry analysis and quantifications of CD4^+^IFNγ.^+^ Th1 cells in mouse brain (**A**), cervical lymph nodes (**B**), ileum (**C**) and colon (**D**). *n* = 5:5:5. Values are expressed as mean ± SEM. Two-way ANOVA followed by Tukey’s multiple comparisons test. ns, not significant. ns, not significant. *P < 0.05, **P < 0.01

We further analyzed the subtype of CD4^+^ T cells by flow cytometry. In brain tissue, an increase in Th1 cells (CD4^+^ IFNγ^+^) was observed in MPTP-induced PD mice compared with control mice, and mice treated with MPTP + LIP-mix showed more Th1 cells than MPTP treated mice (Fig. 5A).

Then, we examined peripheral Th1 cells in the cervical lymph nodes, the ileum and the colon. In cervical lymph nodes and ileum, Th1 cells were significantly increased in the MPTP + LIP-mix group compared with the MPTP group, although there was no difference between the control group and the MPTP group (Fig. 5B, C). Notably, the number of Th1 T cells in the colon was increased in the MPTP group compared with the control group, and LIP-mix treatment further stimulated Th1 cell accumulation in MPTP-induced PD mice (Fig. 5D).

### IFNγ neutralization alleviates dopaminergic neuronal loss in MPTP + LIP-mix treated mice

**Fig. 6:**
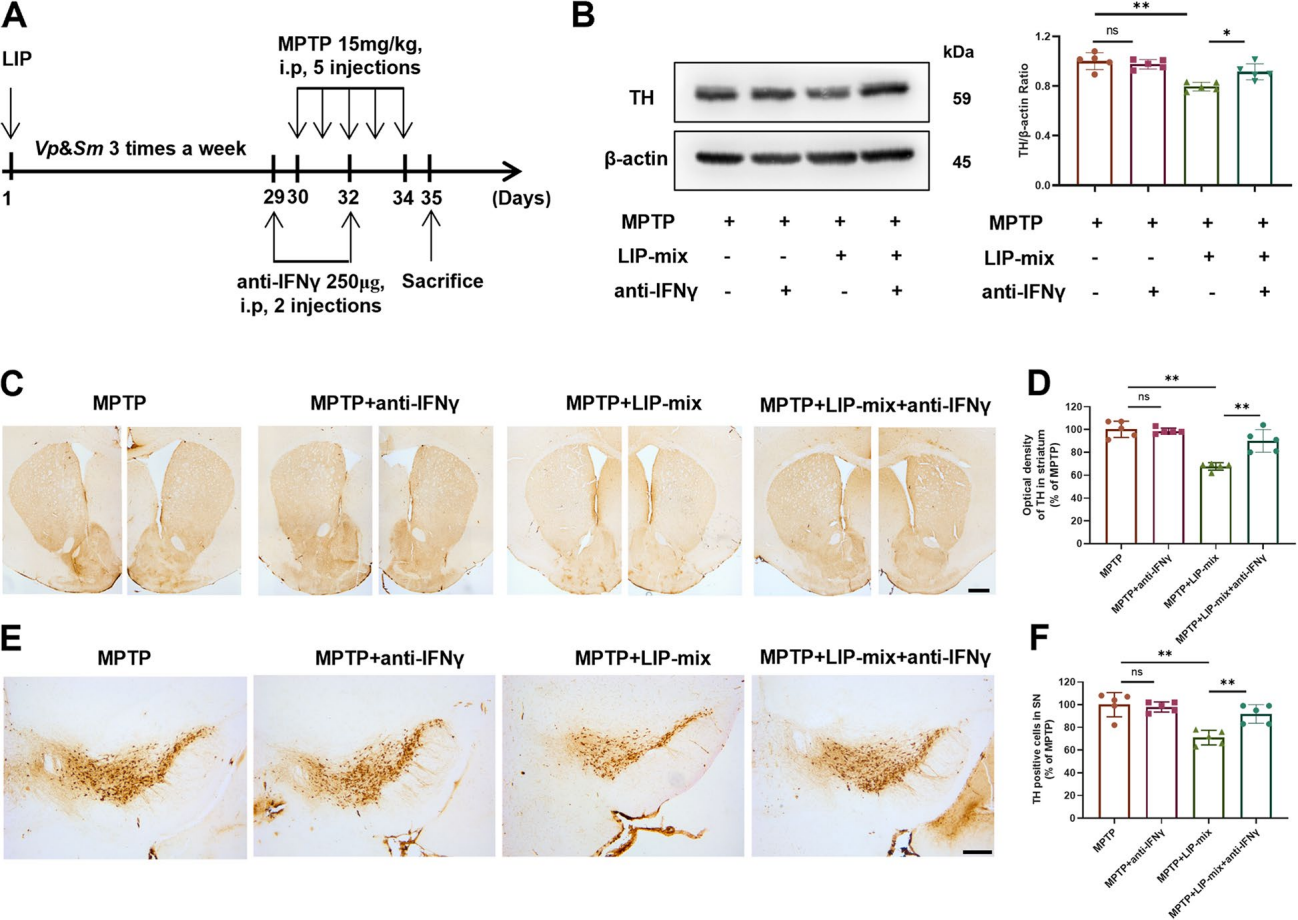
Neutralization of IFNγ alleviates dopaminergic neuronal loss in MPTP + LIP-mix treated mice. **A** Schematic illustration of the experimental procedure. Anti-IFNγ, IFNγ-neutralizing antibody. **B** Western blotting analysis of TH in ST. **C-D**) Representative immunohistochemical staining (**C**) and quantification (**D**) of TH-positive fibers in ST. **E–F** Representative immunohistochemical staining (**E**) and quantification (**F**) of TH-positive neurons in SN. *n* = 5:5:5:5. Scale bar: 200 μm. Values are expressed as mean ± SEM. Two-way ANOVA followed by Tukey’s multiple comparisons test was used for statistical analysis. ns, not significant. *P < 0.05, **P < 0.01

As IFNγ is the main cytokine secreted by Th1 cells, we used an IFNγ neutralizing antibody to verify whether Th1 cells play a critical role in the exacerbation of dopaminergic neuronal loss (Fig. 6A). Compared with that in the MPTP group, the level of TH-positive protein in the ST was significantly decreased in the MPTP + LIP-mix group (Fig. 6B). IFNγ neutralization did not prevent the loss of TH-positive protein loss in MPTP treated mice but alleviated the reduction in TH-positive protein reduction in MPTP + LIP-mix treated mice (Fig. 6B). Similarly, the IHC results showed that LIP-mix exacerbated dopaminergic fiber and neuron loss in the ST and SN of MPTP-treated mice (Fig. 6C-F). Neutralizing IFNγ did not have an effect on MPTP treated mice but attenuated neuronal loss in MPTP + LIP-mix treated mice (Fig. 6C**-**F).

## Discussion

Several clinical studies have substantiated a potential link between periodontitis and PD [14–18]. However, there is a lack of verification and mechanistic explorations in animal models. Here, we demonstrated that within the context of periodontitis, oral pathogens exacerbated motor dysfunction and neurodegeneration in MPTP-induced PD mice by causing oral and gut dysbiosis, as well as brain and systemic immune activation. During this process, the oral pathogens *V. parvula* and *S. mutans* played a key role.

We have shown that LIP-SP promotes the progression of PD. In our model, we used SP to simulate the oral environment of patients with periodontitis [21] and found that LIP-SP exacerbated motor dysfunction and dopaminergic neuron loss and promoted microglial activation. Neuroinflammation is part of the pathophysiological pathway of PD [29]. Previous studies have provided important evidence of microglial activation and proinflammatory cytokine upregulation in MPTP-induced PD mice [2, 30]. Oral *P. gingivalis* infection in mice induces neuroinflammation in other neurodegenerative diseases [20], suggesting that periodontitis is related to neurological diseases.

Oral pathogens play a key role in periodontitis-mediated exacerbation of PD. In the present study, LIP-SP induced oral and gut dysbiosis but failed to change the composition of the microflora in the brain. Previous studies have shown that people with PD have altered oral and gut microbiomes [16, 31]. The gut microbiota is involved in the pathogenesis of PD [32]. For example, germ-free mice that received fecal microbiota transplantation from PD patients exhibited increased motor dysfunction [3], and reestablishing a normal gut microbial community in PD mice alleviated neuroinflammation and dopaminergic neuron loss [26]. There is a connection between the oral and gut microbiomes in patients with PD [33]. Oral dysbiosis can cause systemic effects through local and systemic inflammation and oral-gut communication [33]. Under pathological conditions, oral pathogens can affect gut inflammation and strengthen the connection between oral and gut dysbiosis [27].

*V. parvula* and *S. mutans* exert an exacerbating effect on PD in the context of periodontitis. We found that *V. parvula* and *S. mutans* from SP were enriched in the gut of MPTP + LIP-SP treated mice. It has been reported that *V. parvula* is tightly associated with periodontitis [34]. *S. mutans* is a common oral pathogen and increased in dental plaque of patients with PD [16]. Here, we found that the oral pathogens *V. parvula* and *S. mutans* could exacerbate motor dysfunction and TH^+^ neuron loss in MPTP-induced PD mice. Previous studies have shown that *P. gingivalis* can exacerbate dopaminergic neuron loss in PD mice by causing gut dysbiosis and elevating peripheral inflammatory cytokines [19]. These findings suggest that there may be more oral pathogens that affect PD and have yet to be discovered.

The cerebral and peripheral immune activation may be the mechanism by which *V. parvula* and *S. mutans* exacerbate PD in the context of periodontitis. Neuroinflammation plays an important role in PD progression. In this study, *V. parvula* and *S. mutans* significantly promoted microglial activation and CD4^+^ T cell accumulation in the brains of MPTP-induced PD mice. Compared with that of healthy people, the number of CD4^+^ T cells in the substantia nigra of PD patients is increased, and CD4^+^ T cells play an important role in the neurodegeneration of PD [35, 36]. It has been reported that periodontitis induces the accumulation of a large number of immune cells in cervical lymph nodes, and that these immune cells can transmigrate to other organs, including the gut [37]. Periodontal pathogens can migrate through the digestive tract and ectopically colonize in the gut. Oral pathobiont-reactive T cells can transmigrate to the gut, where they are activated by the gut microbiota and induce gut inflammation [27]. Our study also showed that *V. parvula* and *S. mutans* gave rise to the massive production of CD4^+^ T cells in cervical lymph nodes, the ileum and the colon. These tissues may be the sources of CD4^+^ T cells in the brains of PD mice. A previous study showed that cervical lymph nodes were the source of CD4^+^ T cells in the PD brain [38]. However, whether gut CD4^+^ T cells can migrate to the brain in PD needs further exploration.

*V. parvula* and *S. mutans* worsen PD by promoting the production of Th1 cells. In the present study, *V. parvula* and *S. mutans* accelerated Th1 cell infiltration in the brain, cervical lymph nodes, and the gut in MPTP-induced PD mice. Naïve CD4^+^ T cells in the peripheral blood of PD patients have a tendency to differentiate into Th1 cells [39]. Regarding the role of Th1 cells in PD, a previous study has shown that adoptive transfer of Th1 cells into MPTP-induced PD mice increased the degree of neurodegeneration [40]. Th1 cells are associated with the activation of myeloid cells in the CNS [36]. The effect of Th1 cells on the CNS and the results of our study together suggest that Th1 cells play an important role in *V. parvula-* and *S. mutans-*induced neurodegeneration. The IFNγ-neutralizing experiment in MPTP + LIP-mix mice showed the importance of IFNγ in our study. IFNγ, the major proinflammatory factor secreted by Th1 cells, can activate microglia to mediate neuroinflammation [41, 42]. The role of IFNγ in MPTP-induced PD is controversial. When the MPTP dose was 25 mg/kg, IFNγ knockout alleviated MPTP-induced dopaminergic neuron loss [42]. When the MPTP dose was 20 mg/kg, IFNγ knockout did not play a protective role in MPTP-induced PD [35]. In the present study, we used 15 mg/kg MPTP to induce PD [43], which may explain why the IFNγ-neutralizing antibody had no effect on MPTP-induced PD mice without the simulation by LIP-mix.

## Conclusions

Overall, oral pathogens *V. parvula* and *S. mutans* exacerbate the severity of PD in the context of periodontitis. Mechanistically, *V. parvula* and *S. mutans* alter the oral and gut microbiota and induce Th1 cell infiltration in both peripheral and brain regions, thereby intensifying microglial activation and contributing to neurodegeneration. Our study unravels the mechanism through which oral pathogens exacerbate PD, offering a novel direction for the prevention and treatment of PD.

### Supplementary Information

**Additional file 1:** **Supplementary Figure S1.** Periodontitis caused severe alveolar bone resorption in mice. LIP, ligature-induced periodontitis. SP, subgingival plaque. LIP-SP, ligature-induced periodontitis with application of subgingival plaque. LIP-mix: ligature-induced periodontitis and oral infection of *V. parvula *and *S. mutans* mixture. (A) Representative images of left maxilla from the indicated groups of mice. (B) Quantification of the distance from cementoenamel junction (CEJ) to alveolar bone crest (ABC). Values are expressed as mean ± SEM (standard error of the mean). ns, not significant, **P < 0.01. **Supplementary Figure S2.** Neither LIP nor SP alone could aggravate dopaminergic neuronal loss in MPTP-induced PD mice. (A**-**B) Representative immunohistochemical staining (A) and quantification (B) of TH-positive fibers in ST. (C**-**D) Representative immunohistochemical staining (C) and quantification (D) of TH-positive neurons in SN. Scale bar: 200 μm. (E-F) Western blotting analysis of TH in ST. Values are expressed as mean ± SEM. ns, not significant. **Supplementary Figure S3.** Pathogens from subgingival plaques are not detected in the brains of MPTP + LIP-SP treated mice. (A) Alpha diversity of brain microbiota assessed by Chao 1, Faith’s pd, and Shannon index. (B) Beta diversity of brain microbiota. (C) Random forest analysis of brain microbiota at the species level. No pathogens from subgingival plaques were detected in the brains of MPTP + LIP-SP group. **Supplementary Figure S4.** Oral administration of *V. parvula* and* S. mutans *mixture (mix) does not result in bacterial colonization and exacerbation of MPTP-induced PD in mice. (A) Schematic illustration of the experimental procedure. Mix, *V. parvula* and* S. mutans* mixture. (B) Quantitative real-time polymerase chain reaction (qRT**-**PCR) analysis of *Veillonella parvula* and *Streptococcus mutans* in feces. *n*=3:3. (C) Western blotting analysis of TH in ST. Values are expressed as mean ± SEM. ns, not significant. *P < 0.05.

## Data Availability

The data that support the findings of this study are openly available in NCBI, reference number PRJNA944412.

## Abbreviations

PD: Parkinson’s disease
LIP: Ligature-induced periodontitis
SP: Subgingival plaque
LIP-SP: Ligature-induced periodontitis with application of subgingival plaque
*V. parvula*: *Veillonella parvula*; *S. mutans*: *Streptococcus mutans*
Mix: *V. parvula* and *S. mutans* mixture
LIP-mix: Ligature-induced periodontitis and oral infection of *V. parvula* and *S. mutans* mixture
MPTP: 1-Methyl-4-phenyl-1,2,3,6-tetrahydropyridine
OCT: Optimal cutting temperature compound
TH: Tyrosine hydroxylase
ST: Striatum
SN: Substantia nigra
CNS: Central nervous system
DAPI: 4’,6-Diamidino-2-phenylindole dihydrochloride
