# Supplementary material for: Oral pathogens exacerbate Parkinson’s disease by promoting Th1 cell infiltration in mice
Source: Microbiome. 2023 Nov 17;11:254. doi: 10.1186/s40168-023-01685-w (PMC10655362; doi:10.1186/s40168-023-01685-w)
Supplement: Supplementary file 2 — Additional file 1: Supplementary Figure S1. Periodontitis caused severe alveolar bone resorption in mice. LIP, ligature-induced periodontitis. SP, subgingival plaque. LIP-SP, ligature-induced periodontitis with application of subgingival plaque. LIP-mix: ligature-induced periodontitis and oral infection of V. parvula and S. mutans mixture. (A) Representative images of left maxilla from the indicated groups of mice. (B) Quantification of the distance from cementoenamel junction (CEJ) to alveolar bone crest (ABC). Values are expressed as mean ± SEM (standard error of the mean). ns, not significant, **P < 0.01. Supplementary Figure S2. Neither LIP nor SP alone could aggravate dopaminergic neuronal loss in MPTP-induced PD mice. (A-B) Representative immunohistochemical staining (A) and quantification (B) of TH-positive fibers in ST. (C-D) Representative immunohistochemical staining (C) and quantification (D) of TH-positive neurons in SN. Scale bar: 200 μm. (E-F) Western blotting analysis of TH in ST. Values are expressed as mean ± SEM. ns, not significant. Supplementary Figure S3. Pathogens from subgingival plaques are not detected in the brains of MPTP + LIP-SP treated mice. (A) Alpha diversity of brain microbiota assessed by Chao 1, Faith’s pd, and Shannon index. (B) Beta diversity of brain microbiota. (C) Random forest analysis of brain microbiota at the species level. No pathogens from subgingival plaques were detected in the brains of MPTP + LIP-SP group. Supplementary Figure S4. Oral administration of V. parvula and S. mutans mixture (mix) does not result in bacterial colonization and exacerbation of MPTP-induced PD in mice. (A) Schematic illustration of the experimental procedure. Mix, V. parvula and S. mutans mixture. (B) Quantitative real-time polymerase chain reaction (qRT-PCR) analysis of Veillonella parvula and Streptococcus mutans in feces. n=3:3. (C) Western blotting analysis of TH in ST. Values are expressed as mean ± SEM. ns, not significant. *P < 0.05. [file 40168_2023_1685_MOESM1_ESM.docx]

# Supplementary information


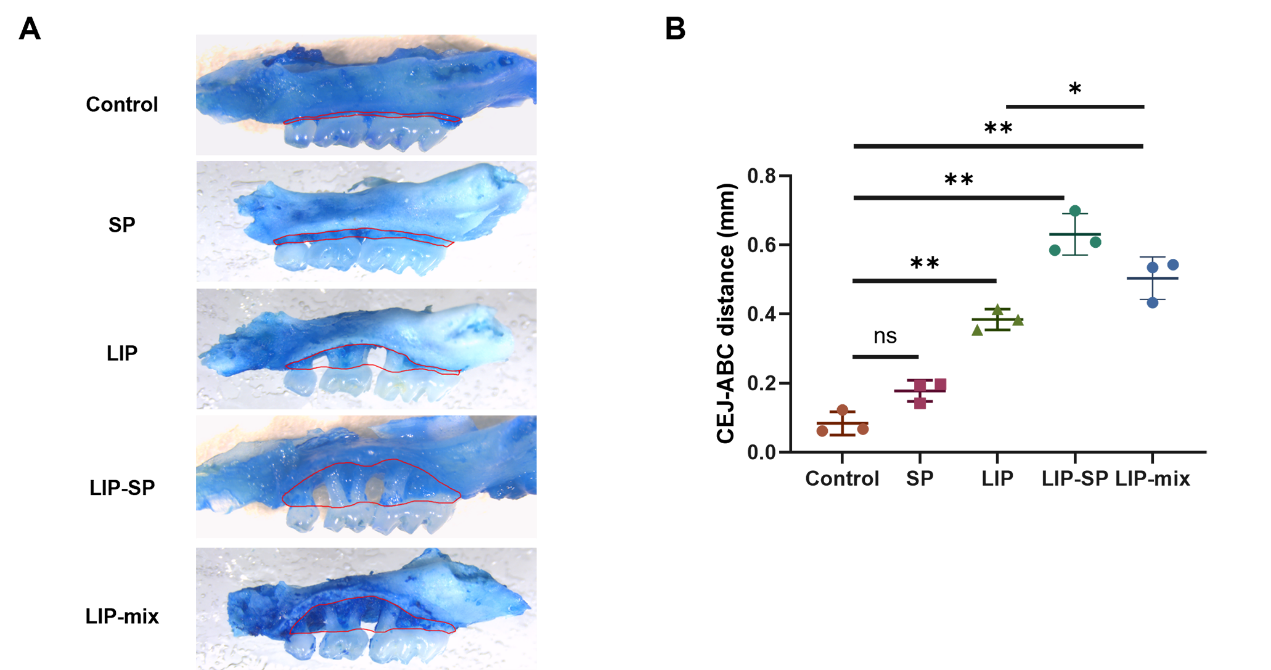


**Figure S1. Periodontitis caused severe alveolar bone resorption in mice.**  LIP, ligature-induced periodontitis. SP, subgingival plaque. LIP-SP, ligature-induced periodontitis with application of subgingival plaque. LIP-mix: ligature-induced periodontitis and oral infection of *V. parvula* and *S. mutans* mixture. (A) Representative images of left maxilla from the indicated groups of mice. (B) Quantification of the distance from cementoenamel junction (CEJ) to alveolar bone crest (ABC). Values are expressed as mean ± SEM (standard error of the mean). ns, not significant, **P < 0.01.


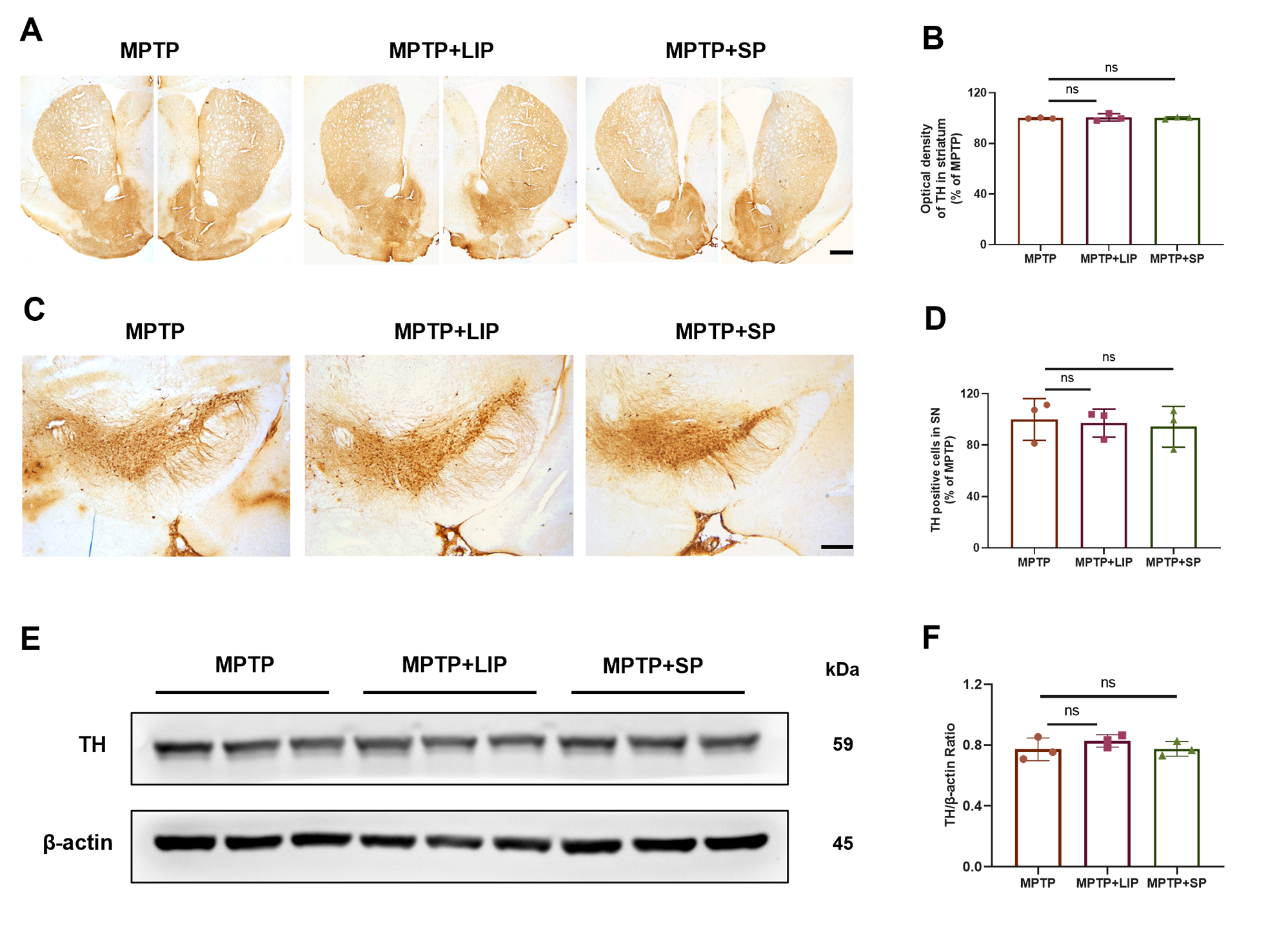


**Figure S2.** **Neither LIP nor SP alone could** **aggravate** **dopaminergic neuronal loss in** **MPTP-induced PD mice.** (A-B) Representative immunohistochemical staining (A) and quantification (B) of TH-positive fibers in ST. (C-D) Representative immunohistochemical staining (C) and quantification (D) of TH-positive neurons in SN. Scale bar: 200 μm. (E-F) Western blotting analysis of TH in ST. Values are expressed as mean ± SEM. ns, not significant.


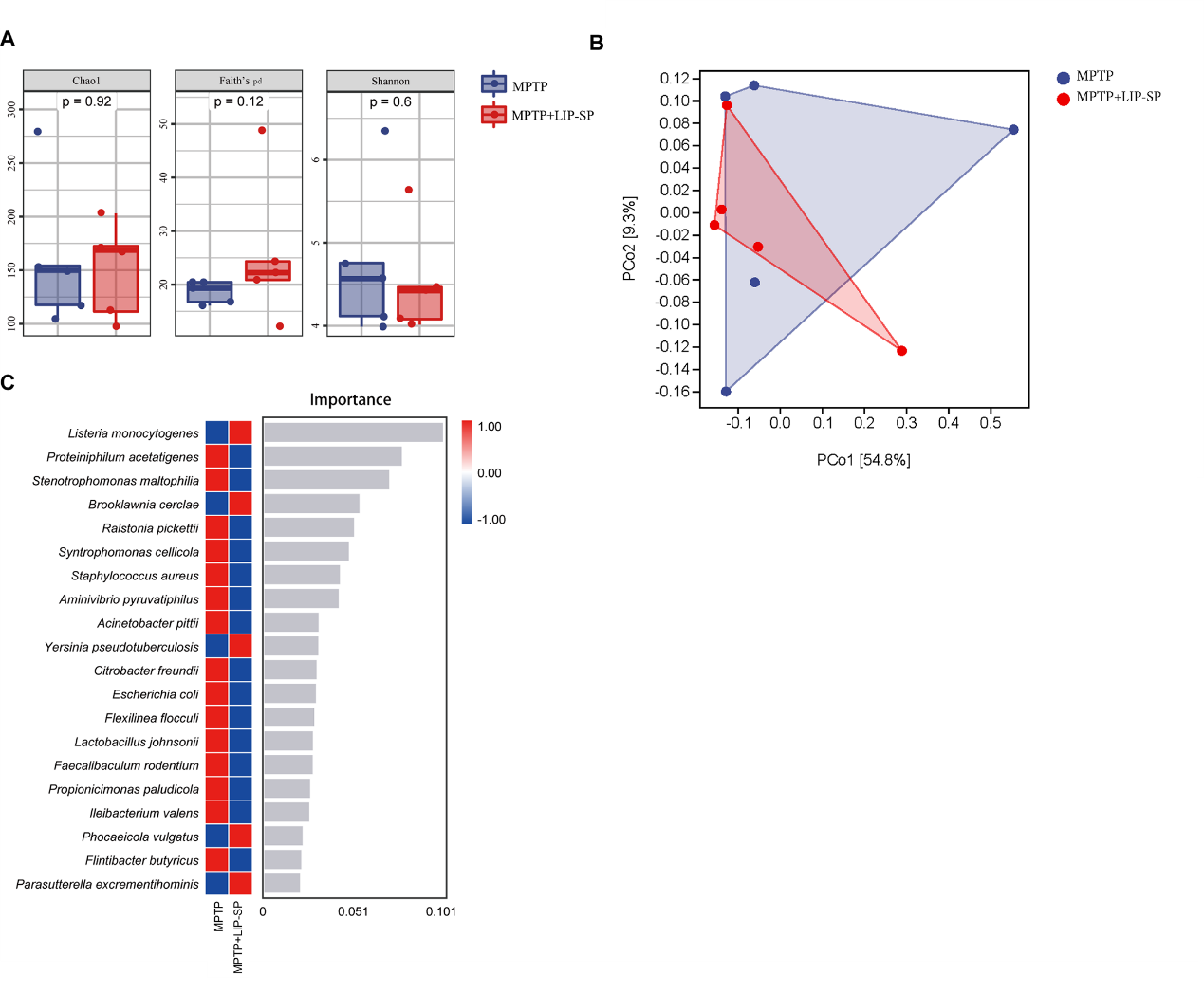


**Figure S3. Pathogens from subgingival plaques are not detected in the brains of MPTP+LIP-SP treated mice.** (A) Alpha diversity of brain microbiota assessed by Chao 1, Faith’s pd, and Shannon index. (B) Beta diversity of brain microbiota. (C) Random forest analysis of brain microbiota at the species level. No pathogens from subgingival plaques were detected in the brains of MPTP+LIP-SP group.


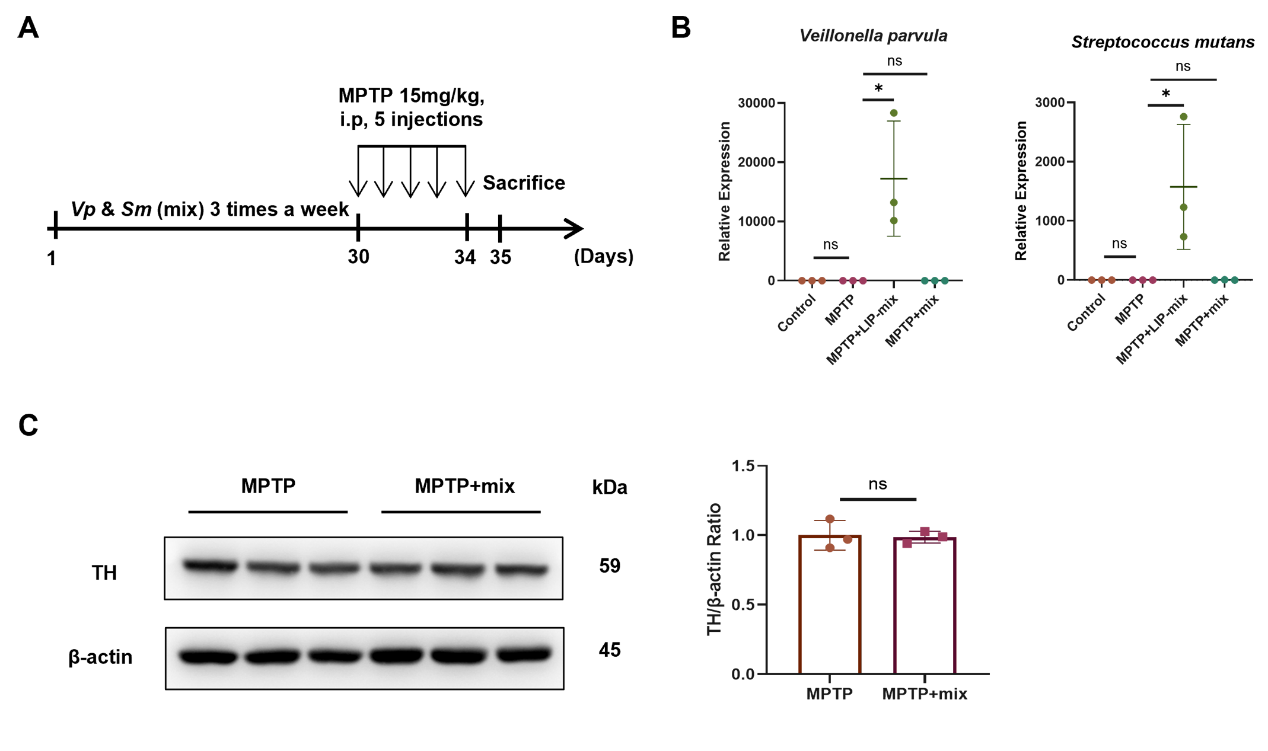


**Figure S4. Oral administration of *V. parvula* and *S. mutans* mixture (mix) does not result in bacterial colonization and exacerbation of MPTP-induced PD in mice.** (A) Schematic illustration of the experimental procedure. Mix, *V. parvula* and *S. mutans* mixture. (B) Quantitative real-time polymerase chain reaction (qRT-PCR) analysis of *Veillonella parvula* and *Streptococcus mutans* in feces. *n*=3:3. (C) Western blotting analysis of TH in ST. Values are expressed as mean ± SEM. ns, not significant. *P < 0.05.

**Materials and Methods for 16S-rRNA sequencing**

We extracted total genomic DNA samples from brain tissue, oral ligatures, and fecal samples using the OMEGA Soil DNA Kit (M5635-02) from Omega Bio-Tek in Norcross, GA, USA. PCR amplification of nearly full-length bacterial 16S rRNA genes was carried out using the forward primer 27F (5’-AGAGTTTGATCMTGGCTCAG-3’) and the reverse primer 1492R (5’-ACCTTGTTACGACTT-3’). During the two-step PCR amplification process, sample-specific 16-bp barcodes were incorporated into both the forward and reverse primers for subsequent multiplex sequencing in the second PCR step. Each PCR reaction consisted of 5 μl of Q5 reaction buffer (5×), 5 μl of Q5 High-Fidelity GC buffer (5×), 0.25 μl of Q5 High-Fidelity DNA Polymerase (5U/μl), 2 μl (2.5 mM) of dNTPs, 1 μl (10 uM) of each forward and reverse primer, 2 μl of DNA template, and 8.75 μl of ddH2O. The thermal cycling program involved an initial denaturation at 98 °C for 2 minutes, followed by 25 cycles (for the first amplification step) and 10 cycles (for the second amplification step). Each cycle included denaturation at 98 °C for 30 seconds, annealing at 55 °C for 30 seconds, and extension at 72 °C for 90 seconds, with a final extension step at 72 °C for 5 minutes. The resulting PCR amplicons were purified using Agencourt AMPure Beads from Beckman Coulter in Indianapolis, IN, and quantified using the PicoGreen dsDNA Assay Kit from Invitrogen in Carlsbad, CA, USA.

We utilized Single Molecule Real Time (SMRT) sequencing technology on the PacBio Sequel platform, which was conducted at Shanghai Personal Biotechnology Co., Ltd in Shanghai, China. To minimize sequencing errors, we employed PacBio Circular Consensus Sequencing (CCS) reads derived from multiple alignments of sub-reads. In CCS, the DNA polymerase reads a ligated circular DNA template multiple times, allowing us to effectively generate a consensus sequence from multiple reads of a single molecule. The initial processing of raw sequences was performed through the PacBio SMRT Link portal (version 5.0.1.9585). We filtered the sequences to ensure a minimum of 3 passes and a minimum predicted accuracy of 99% (minfullpass = 3, minPredictedAccuracy = 99). Here, the predicted accuracy of 99% serves as the threshold below which a CCS read is considered noise. Subsequently, files generated by the PacBio platform underwent amplicon size trimming to remove sequences exceeding 2,000 base pairs in length.

We conducted microbiome bioinformatics analyses using QIIME2 version 2019.4, with minor adjustments based on the official tutorials available at (https://docs.qiime2.org/2019.4/tutorials/). The initial processing of raw sequence data included demultiplexing using the demux plugin, followed by primer trimming using the cutadapt plugin^1^. Subsequently, the sequences underwent a series of steps, including quality filtering, denoising, merging, and chimera removal, which were executed using the DADA2 plugin^2^ to obtain amplicon sequence variants (ASVs) that were not singletons. These ASVs were then aligned using mafft^3^ and utilized to construct a phylogeny with fasttree2^4^.

We conducted sequence data analyses using a combination of QIIME2 and R packages (v3.2.0). Alpha diversity indices at the ASV level, including the Chao1 richness estimator, Shannon diversity index, and Faith’s index, were computed using the ASV table within QIIME2. To facilitate visualization, these indices were represented as box plots. For beta diversity analysis, we assessed the structural variation of microbial communities across samples. Principal Coordinate Analysis (PCoA) was utilized to visualize the results. To distinguish samples from different groups, we employed Random Forest analysis within QIIME2, utilizing the default settings.

**References of supplementary information**

1. Martin M. Cutadapt removes adapter sequences from high-throughput sequencing reads. *EMBnet*. 2011; 17(1): pp–10.

2. Callahan BJ, Mcmurdie PJ, Rosen MJ, *et al*. Dada2: high-resolution sample inference from illumina amplicon data. *Nature Methods*. 2016; 13(7), 581-583.

3. Katoh K, Misawa K, Kuma K, *et al*. Mafft: a novel method for rapid multiple sequence alignment based on fast fourier transform. *Nucleic Acids Research*. 2002; 30(14), 3059-3066.

4. Price MN, Dehal PS, Arkin AP. FastTree: computing large minimum evolution trees with profiles instead of a distance matrix. *Mol Biol Evol*. 2009; 26(7):1641-1650.
